# A Cost-Effective In Situ Zooplankton Monitoring System Based on Novel Illumination Optimization

**DOI:** 10.3390/s20123471

**Published:** 2020-06-19

**Authors:** Zhiqiang Du, Chunlei Xia, Longwen Fu, Nan Zhang, Bowei Li, Jinming Song, Lingxin Chen

**Affiliations:** 1 CAS Key Laboratory of Marine Ecology and Environmental Sciences, Institute of Oceanology, Chinese Academy of Sciences, Qingdao 266071, China; zqdu@yic.ac.cn (Z.D.); jmsong@qdio.ac.cn (J.S.); lxchen@yic.ac.cn (L.C.); 2 CAS Key Laboratory of Coastal Environmental Processes and Ecological Remediation, Research Center for Coastal Environmental Engineering and Technology, Yantai Institute of Coastal Zone Research, Chinese Academy of Sciences, Yantai 264003, China; lwfu@yic.ac.cn (L.F.); ylopj@vip.sina.com (N.Z.); bwli@yic.ac.cn (B.L.); 3 Center for Ocean Mega-Science, Chinese Academy of Sciences, Qingdao 266071, China; 4 Laboratory for Marine Ecology and Environmental Science, Pilot National Laboratory for Marine Science and Technology (Qingdao), Qingdao 266237, China

**Keywords:** zooplankton monitoring, microscopic imaging, dark-field imaging, illumination optimization, genetic algorithm

## Abstract

A cost-effective and low-power-consumption underwater microscopic imaging system was developed to capture high-resolution zooplankton images in real-time. In this work, dark-field imaging was adopted to reduce backscattering and background noise. To produce an accurate illumination, a novel illumination optimization scheme for the light-emitting diode (LED) array was proposed and applied to design a lighting system for the underwater optical imaging of zooplankton. A multiple objective genetic algorithm was utilized to find the best location of the LED array, which resulted in the specific illumination level and most homogeneous irradiance in the target area. The zooplankton imaging system developed with the optimal configuration of LEDs was tested with *Daphnia magna* under laboratory conditions. The maximal field of view was 16 mm × 13 mm and the optical resolution was 15 μm. The experimental results showed that the imaging system developed could capture high-resolution and high-definition images of *Daphnia*. Subsequently, *Daphnia* individuals were accurately segmented and their geometrical characters were measured by using a classical image processing algorithm. This work provides a cost-effective zooplankton measuring system based on an optimization illumination configuration of an LED array, which has a great potential for minimizing the investment and operating costs associated with long-term in situ monitoring of the physiological state and population conditions of zooplankton.

## 1. Introduction

Zooplankton play an important role in aquatic ecosystems and are the foundation of the food web [1]. They are useful indicators of the future health of fisheries because they are a food source for organisms at higher trophic levels and are thus of interest to oceanographic researchers. In the past, traditional techniques (such as filters, pumps, and nets) for investigating the abundance of zooplankton were laborious and time-consuming [2,3]. Additionally, many zooplankton are fragile gelatinous organisms that are damaged or destroyed by nets and pumps. Therefore, optical imaging instruments have been developed for the investigation of phytoplankton, zooplankton, marine snow particles, and metazoans [4,5], including the Video Plankton Recorder (VPR) [6,7], Underwater Vision Profiler (UVP) [8], ZOOplankton VISualization system (ZOOVIS) [9], Light frame On-sight Key species Investigate System (LOKI) [10,11], Shadow Image Particle Profiling Evaluation Recorder (SIPPER) [12], and In Situ Ichthyoplankton Imaging System (ISIIS) [13]. These imaging instruments can provide images to the dedicated data processing systems for further data analysis. Because many zooplankton are transparent or translucent and have a variety of shapes and surfaces, incident light can be transmitted, reflected, scattered, or absorbed by the various tissues of an organism [14]. The orientation of the lamp to the camera and field of view dictate the area illuminated and image contrast, which directly affect the perception of objects’ details, including the surface texture, shape, and transparency. For example, the previous version of the SIPPER instrument often encountered the problem of artifacts during real-time image segmentation [15]. Since the light beam was more intense near its center, the algorithm based on the binarization threshold resulted in image artifacts of transparent particles near the edges of the beam. At the same time, small defects or debris on the optical windows also resulted in constant dark pixels, which were capable of causing false recognition. The artifacts could be removed with computer post-processing, but this decreased the real-time performance of the system in processing data.

With the development of optical imaging systems, the appropriate design of illumination has become more and more important for keeping pace with the highly developed image acquisition techniques. In order to obtain a better light homogeneity in the field of view, the two lighting units of the Underwater Video Profiler (UVP) were fixed on an aluminum plate facing each other. In UVP, 42 LEDs were set behind two semi-cylindrical lenses to generate a uniform light field. Bright LEDs can produce a sharp contrast image in an underwater environment. A high brightness is essential for avoiding motion blurring of the particles during exposure. However, high-power light sources may lead to an inhomogeneous brightness across the field of view and scattering samples can cast ‘shadows’ in the lateral direction. Therefore, illumination homogeneity is also considered for obtaining high-quality super resolution images. It can be clearly seen that illumination has a huge impact on the success of extracting target features from images.

All reported zooplankton imaging systems have two common characteristics, which are their relatively large size and high power consumption [16]. In particular, the underwater illumination relies on a xenon bulb, laser, or many LED light sources, which require a large power supply. Most current instruments are not amenable to small, extended duration autonomous vehicles such as gliders and floats. There are many additional constraints when building zooplankton imaging devices that can sustain in situ measurements for extended periods of time, including the need for low-power, small-mass, and small-volume instruments. Correspondingly, underwater illumination systems are always limited by cost, available space, and thermal problems. Therefore, obtaining the best illumination dimensioning is indispensable for achieving adequate lighting and a reduced electrical power consumption, which can also simplify image processing algorithms and improve the robustness of identification algorithms [17,18].

The motivation of this work is to optimize the design of an underwater illumination device for in situ microscopic imaging. This is one of the most important steps in developing a zooplankton imaging system. The luminance level is generally considered in most applications and is set using the number of LEDs or the size of the source plane. However, the uniformity and light efficiency are frequently neglected. The traditional theoretical calculation method makes it difficult to find the ideal LED array structure. The uniformity, optimal lux level, power efficiency, spatial distribution of the source, and source geometry are interrelated [19]. Therefore, this paper proposes a novel optimization scheme for quickly determining a good solution for designing an underwater illuminator. Furthermore, our main contributions are also developing a simple-structured and cost-effective zooplankton imaging system and demonstrating the performance of obtaining high-resolution images through underwater tests.

Automated zooplankton imaging systems have been studied for decades. The high cost and sophisticated structure have been identified as the main factors hindering the popularization of automatic underwater microscopic imaging in many studies. The proposed underwater microscopic imaging system could efficiently promote the development of in situ biological observation systems for scientific research and its applications.

## 2. Experimental Section

### 2.1. Materials

*Daphnia* were obtained from the local market (Yantai, China) and cultivated in the laboratory, and served as the test organism. The zooplankton imaging system mainly includes the following components: a light source, an image sensor, and image capture and processing hardware. In order to present the physiological state and morphological characteristics of zooplankton, a high-resolution color digital camera (Blackfly S, 500 MPixels) was purchased from FLIR Systems (Richmond, BC, Canada). A bi-telecentric lens (TC23016) was provided by Opto-Engineering (Milano, Italy), for accurately measuring the size of zooplankton. The underwater lighting system should be designed for high-luminous and high-color rendering. The LED light sources (3535 series) were obtained from Samsung (SEOUL, Korea). The micro-controller (Raspberry PI 3B+) was purchased from the Raspberry PI Foundation (Cambridge, UK), and was compact, with low power requirements.

### 2.2. Underwater Opitcal Imaging of Zooplankton

**Figure 1:**
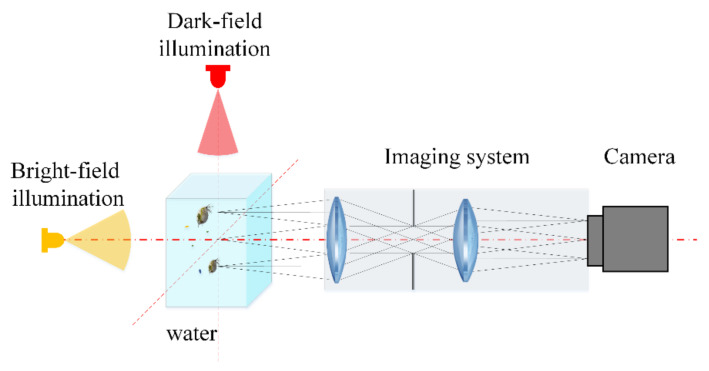
A schematic of the zooplankton imaging system.

Underwater imaging systems with bright-field or dark-field illumination have been used to observe zooplankton in situ, as shown in Figure 1. Bright-field illumination can illuminate the entire sample so that the specimen appears as a dark image against a brightly lit background [20]. However, due to the light scattering caused by the huge number of particles in seawater, the images acquired by a bright-field imaging system usually contain noise, which severely degrades the image quality. It is difficult to separate the partly transparent zooplankton from the background image. Dark-field illumination is a technique employed in optical microscopy that eliminates scattered light from sample images. As a result, only the sample itself is illuminated, while the background remains dark [21]. Therefore, dark-field microscopic imaging was adopted to capture zooplankton images in water. The light sources illuminated the target area from both sides of the optical axis of the imaging system. The digital camera and lens were positioned in front of the target area, while the direction of illumination light was perpendicular to the optical axis. Therefore, illumination light paths and imaging light paths were separated to depress backscattering light delivered to the imaging sensor of the camera. Since the incident light could not directly enter the image optical path, only the scattering beam from the plankton body could be received by the imaging sensor. Consequently, high-contrast and high-definition images of zooplankton could be obtained with a dark background. All objects in the target area were clearly recorded by the camera. Each capture was assumed to examine a water sample equal to the volume of the imaging area. The volume of the target area was determined by the field of view and depth of field of the optical lens.

### 2.3. Design of a Zooplankton Imaging System

**Figure 2:**
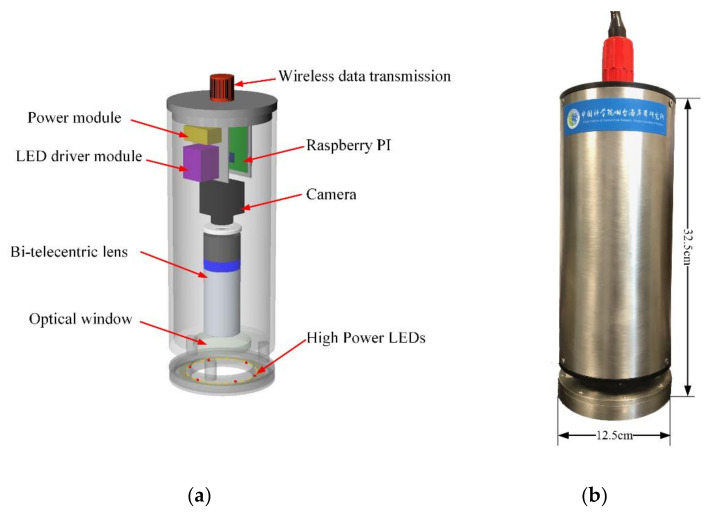
(**a**) The structure of the imaging system; (**b**) a photograph of the imaging system.

The underwater zooplankton imaging system was designed according to the principle of underwater microscopic imaging discussed above. The structure of the imaging system is presented in Figure 2. The micro-controller, digital camera, and optical lens were enclosed in a water-proofed cabinet. Underwater images could be captured by the camera through an optical window. The LED light source was installed in front of the optical window and provided illumination light from outside of the cabinet. To reduce the power consumption and cost of the imaging system, LED lights were chosen as the illumination light source. LED lights were arranged into a ring to provide optimal illumination.

A bilateral telecentric lens was chosen in the proposed imaging system to acquire plankton images with less distortion [22]. In a bilateral telecentric system, the chief rays in the object space and image space are parallel to the optical axis and the magnification is constant within the depth of field, which is essential for removing parallax effects and position-dependent magnification errors, associated with standard lenses. In addition, telecentric lenses have a relatively large depth of field, which enlarges the volume of the water sample at each measurement. The model of the bi-telecentric lens was determined according to the size of plankton and the desired optical resolution. This work aimed to capture mainly zooplankton, which usually has a size ranging from 200 μm to 10 mm. Consequently, the maximal imaging area was determined to be 16 mm × 13 mm in the proposed imaging system. The bi-telecentric lens with the same field of view was selected. The depth of field of the lens was 2 mm and magnification from the sample to the camera sensor was 0.528. The object-side resolution and image-side resolution were 5 and 9 μm, respectively.

Living zooplankton can move rapidly in dynamic water [23]. Therefore, a high-speed camera with a global shutter and short exposure time is necessary to acquire high-definition images of plankton. A digital camera with a 500 megapixel resolution and 75 fps was installed in the proposed image system. The minimal exposure time was set to 1 ms to eliminate motion blurring on images. A low-cost and low-power-consumption single-board computer, Raspberry PI, was chosen as the micro-controller of the imaging system. This controller was in charge of camera control, data collection and transmission, and synchronization between illumination lights and the camera shutter.

The image quality of the microscopic imaging system highly depends on a large amount of light at a high uniformity. The challenge was controlling the illuminance level and uniformity of the illumination area. Therefore, an optimization strategy was studied for designing an enhanced light source with a high intensity and uniform illumination for underwater plankton imaging.

### 2.4. Design of an Optimized Illuminator

#### 2.4.1. Analysis of the Illumination Properties of the LED Array

**Figure 3:**
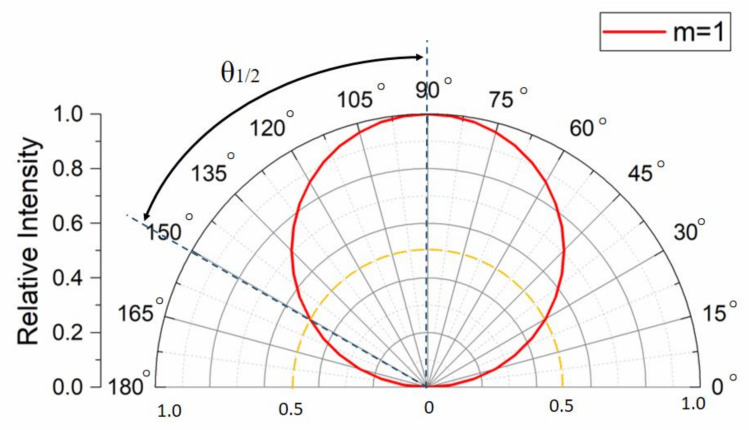
Luminous intensity distribution curve of an ideal Lambertian emitter.

Compared with the traditional light source, light-emitting diodes (LEDs) have many advantages, such as a relatively low energy consumption, long lifetime, and small size, and allow fast switching, which is increasingly being used in underwater illumination applications [24]. Ideally, an LED source is a Lambertian emitter which indicates that the irradiance distribution is a cosine function of the viewing angle. In practice, this dependence is a power law that primarily depends on the encapsulant and semiconductor region shapes. The luminous intensity distribution of LEDs can be expressed as a power function of cosine of the view angle (Equation (1) [25]):

(1) $$I(\theta )=I_{0}\cos^{m}\theta ,$$

where *I*_0_ represents the luminous intensity of the center light-emitting angle, *θ* is the view angle of the LED, and *m* is the parameter indicating the luminous intensity spatial distribution. For example, *m* equals 1 for an ideal Lambertian emitter. The relationship between the emission angles and axial light intensity distribution for the ideal Lambertian emitter is shown in Figure 3. The parameter *m* is calculated using the half width viewing angle *θ*_1*/*2_, which is the viewing angle at which the radiant intensity is half of the value in the normal direction [26], which is given by:

(2) $$m=\frac{-\ln2}{\ln\left(cos\theta_{1/2}\right)}.$$

(1) Illumination Analysis of a Single LED

In practical applications, a single LED is generally considered as a point light source. Therefore, the intensity per unit area varies in inverse proportion to the square of the distance. Additionally, the intensity of the LED light will decrease in water due to light absorption by water. Assuming that the surface element *dS* receives the luminous flux *dφ*, the distance from the LED point light source to the surface element is *l.* The illuminance on the surface element can be formulated by Equation (3):

(3) $$E\left(\theta ,l\right)=\frac{d\varphi }{dS}=\frac{I\left(\theta \right)cos\left(\theta \right)*e^{-c*l}}{l^{2}}$$

where *c* is the attenuation coefficient of the medium, which represents combined light loss in the water.

(2) Illumination Analysis of Group LEDs

Usually, an LED illuminator is composed of a group of LEDs to generate a suitable light intensity and illumination homogeneity, since a single LED cannot provide sufficient illumination. The overall illumination pattern of an LED array is obtained by the superposition of the different illumination patterns of each LED. The illumination pattern of an LED array ($E_{sum}\left(\theta ,l\right)$) is described in the following equation:

(4) $$E_{sum}\left(\theta ,l\right)=\underset{i}{\sum }E_{i}\left(\theta_{i},l_{i}\right)$$

where *E_i_* is the illuminance for the *i*th LED with a corresponding angle *θ_i_* and distance *l_i_*. The illumination uniformity of an LED array can be assessed by calculating irradiance values at a large number of observation points over the imaging plane. The coefficient of variation of the root mean square error *CV(RMSE)* is applied to measure the illumination uniformity of the LED array [26,27]:

(5) $$CV\left(RMSE\right)=\frac{\sigma_{E}}{\bar{E}}$$

where $\sigma_{E}$ is the standard deviation in the target plane and $\bar{E}$ Eis the irradiance average. A smaller value of *CV(RMSE)* represents a higher uniformity.

**Figure 4:**
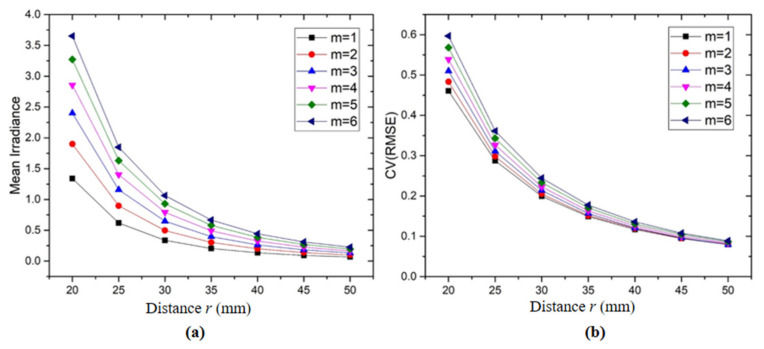
(**a**) The mean irradiance as a function of the distance *r* from a light-emitting diode (LED) lamp to the optical axis at different values of *m*; (**b**) the uniformity (CV(RMSE)) of the illumination surface as a function of the distance *r* from an LED lamp to the optical axis at different values of *m*.

The superposition irradiance of two LEDs arranged symmetrical to the optical axis was analyzed. The two LEDs were equidistant from the center of the optical axis at distance *r*. The distance *r* was set to 20~50 mm, with an interval of 5 mm, and the *m* value of the LED luminous properties ranged from 1 to 6. The mean irradiance and uniformity in the illumination area were presented as a function of the radial distance with different values of *m* (Figure 4). The *x*-axis stands for the distance between the LED and optical axis, and the *y*-axis refers to the mean irradiance or illumination uniformity. The illumination uniformity was calculated by the Equation (5) of *CV(RMSE)*. The average irradiance of the imaging area decreased when the illumination distance increased, while the uniformity of the illumination area increased when increasing the illumination distance. As the radial distance increased, the uniformity characteristics became flatter, indicating that the coverage area became independent of the source’s luminous properties. For example, in the case of *m* = 1, the *CV(RMSE)* of the observation plane decreased from 0.46 to 0.079 when the LED-to-target spacing increased from 20 to 50 mm.

The change in the value *m* of the source had some effect on the illumination surface in the near distance. However, the *CV(RMSE)* of the illumination area at different values of *m* reached nearly 0.08 when the distance from the LED to the optical axis was 50 mm. Therefore, the placement of LEDs was the major factor affecting the mean irradiance and uniformity of illumination in the imaging area. However, the irradiance and uniformity of the imaging area did not consistently decrease with the increasing of the separation between the two LEDs. Although the uniformity could be improved by increasing the source-to-target distance, this could decrease the illuminance intensity of the imaging plane and requires a lighting system with a larger size.

**Figure 5:**
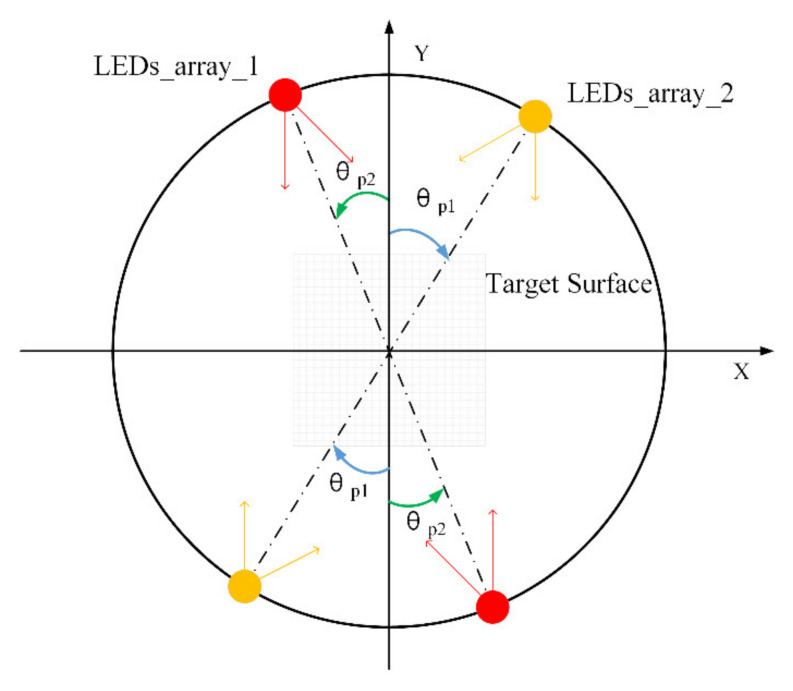
Schematic illustration of two groups of LEDs in a circular arrangement with illumination angle variables *θ_p_*_1_ and *θ_p_*_2_.

The case of two groups of LEDs was subsequently studied at different locations in a circular ring (Figure 5). The positions of the two LEDs of each group were symmetrical about the origin of the coordinates. Two LEDs of each group illuminated the target plane in opposite directions. Each LED with luminous properties *m* = 1 was used in the circular array at a distance of 30 mm from the optical axis. The locations of LEDs were laid out around the center of the imaging plane (i.e., the optical axis). As shown in Figure 5, the illumination angles of the two groups of LEDs are respectively indicated by the variable *θ_p_*_1_ and the variable *θ_p_*_2_. The illumination angle *θ_p_*_1_ of the first group of the LED array was changed in the clockwise direction in the range of 0 to 90°, and the illumination angle *θ_p_*_2_ of the second group of the LED array was changed in the counterclockwise direction in the range of 0 to 90°.

**Figure 6:**
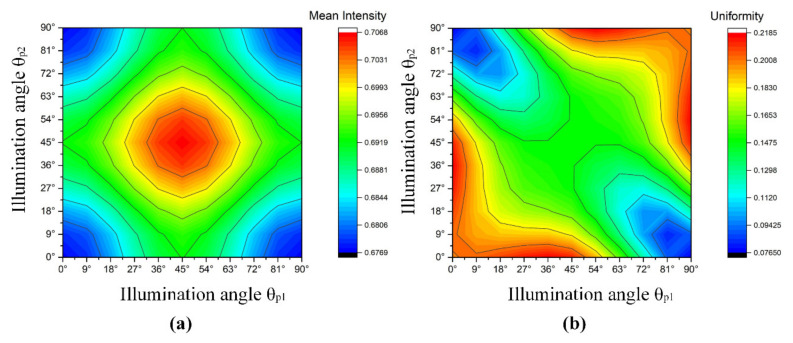
(**a**) Mean irradiance distribution of two groups of LEDs at different illumination angles; (**b**) illumination uniformity distribution of two groups of LEDs at different illumination angles.

Figure 6 shows the mean intensity and uniformity of illumination produced by two pairs of LEDs at different illumination angles. The smallest irradiance was obtained when the pairs of LED arrays were in horizontal or vertical positions. While the two pairs of LED arrays were located at ±45° angles, the largest irradiance value was observed. The ratio of the maximal and minimal mean irradiance in the imaging plane was 1.04.

However, the uniformity of illumination with the LED array in different combinations varied greatly at different locations. The optical structure with the best illumination uniformity was achieved when the two groups of LED arrays were located on the *x*-axis and *y*-axis, respectively, since the combined effect of a number of light sources decreased the variation of irradiance distributions over the target planes. Additionally, the max-min ratio of irradiance uniformity could reach 2.84. This demonstrates that the optimized placement of the LEDs in the array could be an effective scheme for improving the uniformity of the illumination in the imaging area.

Generally, the irradiance pattern depends on the lamps’ locations and the intensity properties. The beams from each LED in the assembly were blended on the imaging plane to produce the total irradiance. Previous works have reported that the inverse square law and Sparrow’s criterion were adopted to obtain uniform illumination [28,29]. According to these works, the angle between each pair of LEDs can be adjusted for investigating optimized individual irradiance patterns. Therefore, the minimum between the maxima from each pair of distributions could be eliminated and the combined irradiance distribution became uniform. However, these methods are suitable for cases in which the light source is parallel to the illumination surface. Although the maximal LED density and the maximal LED-to-target distance may obtain a uniform irradiance distribution over a larger region, the configuration of LED arrays is always limited by many factors, such as the cost, available space, power supply, and thermal problems, in practical applications. Therefore, the optimal parameters for the spatial configuration of an LED array, e.g., the number of LEDs, and the source-target distance in a multi-element LED source or spatial placement of the LEDs, should be determined.

#### 2.4.2. Optimizing the Illumination Configuration by Using the Genetic Algorithm

**Figure 7:**
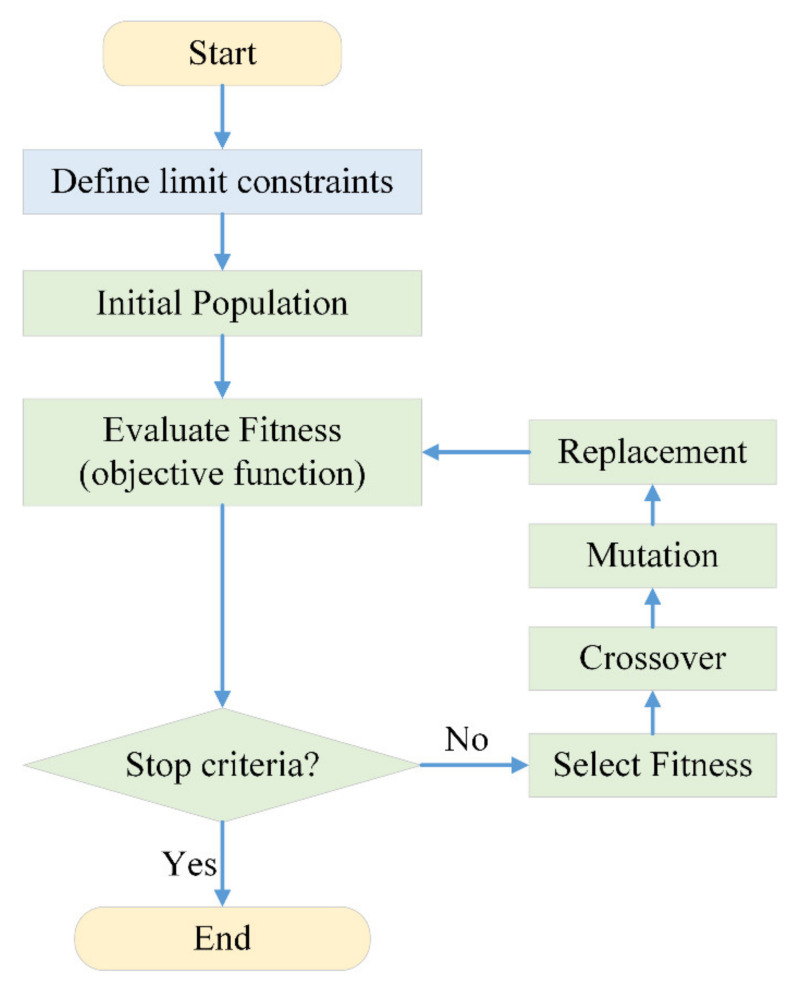
Flow chart of the genetic algorithm.

The genetic algorithm (GA) was applied to optimize the configuration of each LED in the array to produce uniform illumination and an adequate intensity. GA is a kind of searching strategy for global optimization based on genetic evolution and is employed to find a true value by mimicking the process of evolution in natural genetics [30,31]. Compared with other optimization strategies, a benefit of using a GA is the use of optimization with a systematized set of continuous or discrete parameters for global optimization scenarios [32]. A schematic flowchart illustrating the process of the GA is shown in Figure 7. In an optimization problem, each solution candidate is assumed to possess a set of properties (i.e., a chromosome or genotype) in the step of the initial population. A group of random populations in an iteration process is known as a generation. In each generation, the fitness of each individual is evaluated by comparing the value with the objective function of the optimization [33]. In the selection part, multiple population members are chosen at random conditions to create a new generation of offspring based on their fitness functions. The crossover is a process of passing the gene from the parent chromosome to offspring chromosomes. The mutation is a process of mutational genetic modification different from simple genetic inheritance. The replacement is a process that replaces the population with the next generation. The process of fitness evaluation and mutation is conducted again in the new generation, so that a new generation with a higher fitness is obtained. This overall process is repeated until the optimal solution is attained.

The objectives in designing underwater illumination devices are maximizing the performance, maximizing the illumination uniformity, and minimizing the power consumption. The geometry of the LED luminaire, the number of LEDs, their relative positions, and the spatial and spectral distribution of LEDs are usually provided as input parameters to optimize the source configuration. The optimal solution should maximize the overall performance for all objectives [34,35]. The aim of this work was to find the optimal positions for each LED in the LED array to achieve the desired irradiance and maximize the homogeneity of light distribution in the imaging plane. To solve multi-objective optimization problems, the weighted sum method was used to transform a number of objectives into a single objective by assigning weights. The weight of an objective is generally selected in proportion to an objective’s relative importance in the equation. Objectives need to be scaled appropriately to bring them to a similar magnitude. Once the objectives are normalized, a composite objective function can be generated by summing the weighted normalized objectives and is reduced to a single objective optimization problem [36] as follows:

(6) $$min\;z=w_{1}{z_{1}}^{\prime }\left(x\right)+w_{2}{z_{2}}^{\prime }\left(x\right)+\cdots +w_{k}{z_{k}}^{\prime }\left(x\right)$$

The weight vector $w=\left\{w_{1},\;w_{2},\ldots ,\;w_{k}\right\}$ is expected to be provided by the users. The biggest positive feature of the weighted sum approach is its straightforward operation. As a solitary objective is used in fitness allotment, a single objective GA can be used with minor changes. Secondly, this procedure efficiently finds the optimal solution.

Innovative automatically designed systems compare favorably with traditional mathematical methods for designing lighting systems. The following section analyzes the implementation and development of GAs in the optimal design of the lighting system that was considered here.

## 3. Results and Discussion

### 3.1. Design Array Consisting of LEDs Based on the Genetic Algorithm

According to the preliminary tests, the objective irradiance level should reach 15,000 lx (photometric flux per unit area) for capturing images by our imaging system with a 1 ms exposure time. In this section, we optimize the LED array which is made up of six LEDs by the GA algorithm. The implementation of GA in the application of the optimal design of the underwater lighting system incorporates three basic steps: definition of the chromosome structure, and definition of a Fitness Function to evaluate possible solutions and to choose strategic genetic operators.

(1) Chromosomes

A chromosome is composed of various genes and represents a solution to a problem. Each gene represents a variable of the proposed problem. The variables are the angular positions and the radius of the LED ring. In this study, the source was reduced to a plane which was horizontally aligned with the target plane. The range of 0–90° was used for an angular position of each LED. These LEDs were placed around the optical axis in a circle. According to the mechanical limitation, a range of 30–50 mm was given for the radius of the circular array. This reduced the size of the search space by a large extent. Therefore, each variable defined a possible position for a luminaire. The variables in the chromosome were converted into a binary string. The GA started with a random initialization and 20 members in a generation. The search domain of variables was constrained to focus the search effort on specific areas of the feasible region by shrinking the constrained search space.

(2) Fitness function

As explained earlier, the fitness function is a weighting function that measures the quality or performance of a solution for a specific lighting system design. We researched the solution which produced a certain flux level and the most homogeneous lighting on an experimentation plane. In order to search for the optimal position for each lamp in the LED array, a genetic algorithm was mainly used to find the solution for the required irradiance in the first phase. The initialized results were then applied for training data to find the solution to maximize the homogeneity of the light distribution in the second phase. Therefore, an objective function *Φ* was proposed to solve this multi-objective optimization problem in the following equation:

(7) $$\phi =x_{1}+x_{2}\cdot \frac{10}{\left(1+\exp(-x_{2}\cdot 20+8)\right)}$$

where *x*_1_ is the coefficient of variation of the root mean square error of the illumination space, and *x*_2_ is the absolute error between the mean of the intensity levels of the illumination space and the objective irradiance. The exact values of these weighting factors were estimated empirically after rudimental exploration.

The fitness value is the inverse of the objective function. Therefore, a fitness value *φ* for the chromosome can be calculated using the following expression:

(8) $$\varphi =\frac{1}{1+\phi }$$

The target of optimization is to maximize the fitness function or minimize the objective function.

(3) Genetic operators

**Figure 8:**
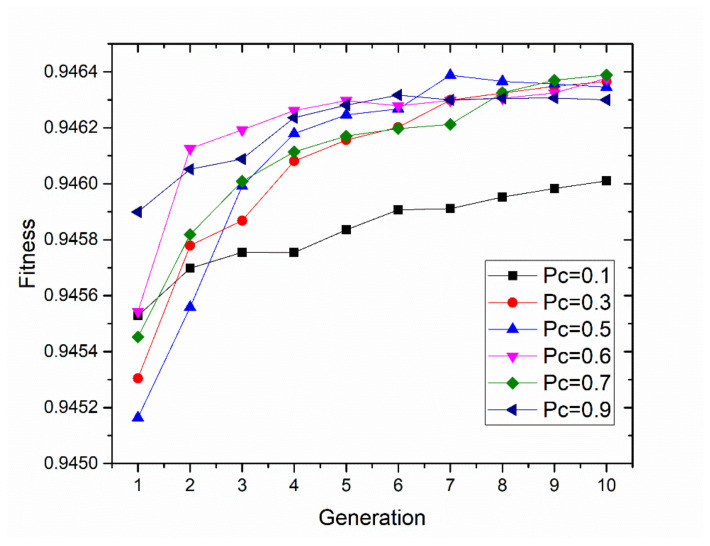
The fitness results at various probabilities of crossover (Pc) from 0.1 to 0.9.

A new generation of child systems is produced from the genetic material of the parent generation. The roulette wheel selection, as a genetic operator, is used for selecting potentially useful solutions for recombination. The mutation operator that was used was the common bit-flipping of each gene of the chromosomes with a specific probability, known as the probability of mutation P_m_, which was also a parameter of exploration. The probability of mutation was set as 0.1. Crossover was performed between couples of the individual parents. A series of experiments were carried out to determine an optimal value range of probability. The crossover’s probability Pc was set in the range from 0.1 to 0.9, and the finishing criterion was set to reach 10 generations. The fitness results and generation values can be observed in Figure 8. The experiment using a 0.6 crossover probability shows a quicker optimal solution for the problem.

**Figure 9:**
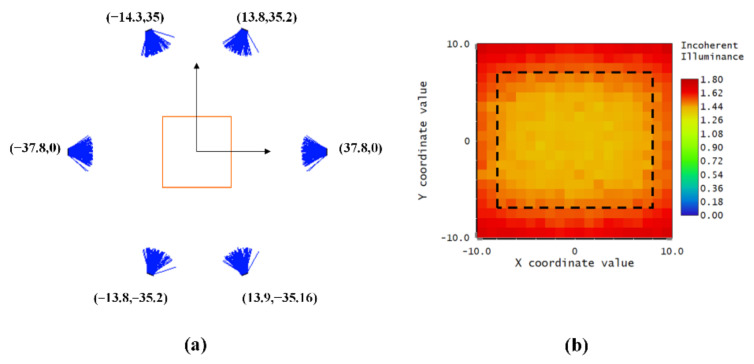
(**a**) The optimized arrangement of the LED array calculated by the genetic algorithm; (**b**) the irradiance distribution over the target plane of the optimized LED array arrangement. Dashed rectangle indicates the imaging area of the camera.

We could build the lighting model with the actual luminescence model of the LED lamp and geometric position of the lamp array, and simulate the light distribution in the whole space, with Monte Carlo ray tracing. In each generation, we simulated the optical system by ray tracing and evaluated the merit function for each member. Optimization of the illumination configuration by GA was achieved after 50 generations. The overall run time was nearly 67 h, since a large number of rays were required to have a stable simulated light pattern in the computation of each member. The simulation result shows that the average irradiance reached 15,000 lx when the distance from LEDs to the optical axis was equal to 37.8 mm. The best uniformity of illumination was achieved when the position coordinates of the six LEDs in the x–y plane were as shown in Figure 9a. The illuminance distribution map of the imaging plane is presented in Figure 9b.

The CV (RSME) reached 0.0553, indicating that the illumination distribution generated by the estimated placement of LED had a high uniformity. These results demonstrated that the proposed scheme is effective for designing the optimized illumination configuration of an LED array. The dashed rectangle marks the field of view of the camera.

### 3.2. Experiments with the Zooplankton Imaging System

**Figure 10:**
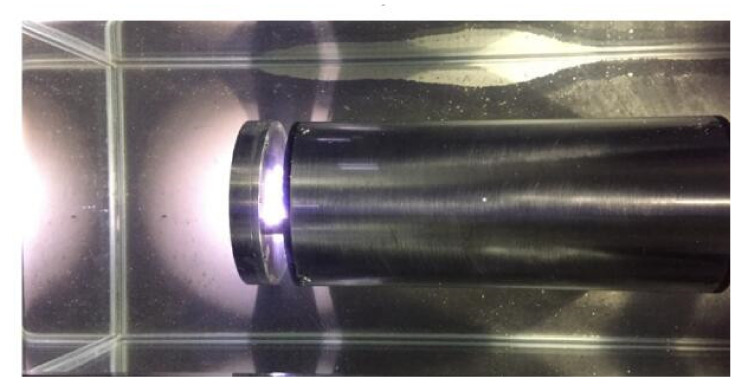
Underwater test for the zooplankton imaging system.

In order to facilitate laboratory testing of the prototype, the zooplankton imaging system was evaluated and tested with a glass tank in the laboratory (Figure 10). The imaging system and live zooplankton samples were placed in the water of a glass tank (60 cm × 18 cm × 28 cm). The sampling software allowed the user to have full control of the system parameters, and the pictures were saved and displayed on the screen.

**Figure 11:**
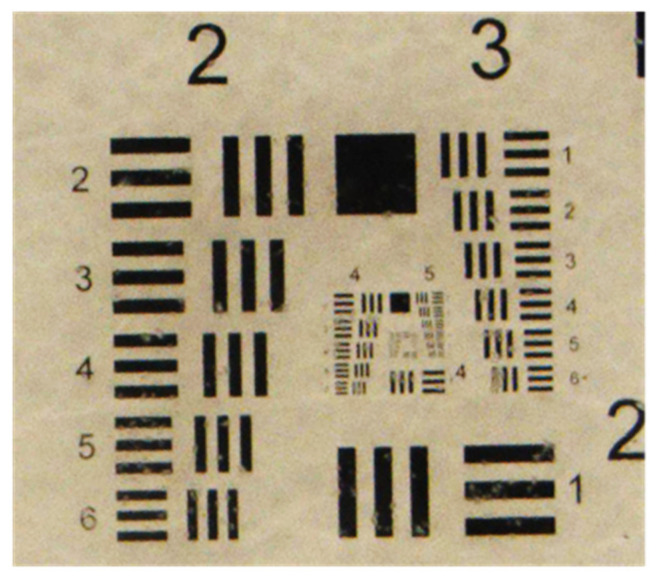
The resolution test picture captured underwater by the system.

The imaging performance was initially tested on a calibrated resolution target slide (1951 USAF resolution target) [37]. The resolution target slide was aligned to the center of the ring of the LED array. The resolution was defined by the frequency measured in line pairs per millimeter (lp/mm). As shown in Figure 11, the optical resolution of the developed underwater microscopic imaging system was determined to be approximately 32 lp/mm. The actual resolution of the underwater optical imaging system was approximately 15 μm. Therefore, the resolution and field of view of the plankton imaging system could satisfy the requirements for observing medium and large zooplankton.

**Figure 12:**
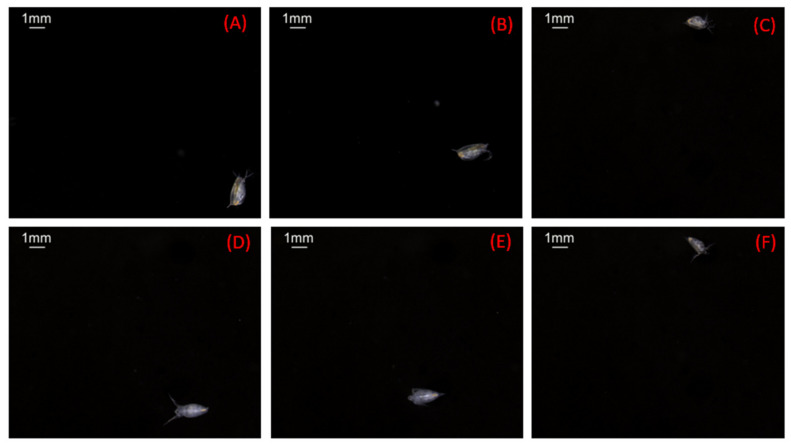
Images of zooplankton captured by the imaging system in the water (**A**–**F**).

Subsequently, underwater imaging tests were conducted on living biological organisms. *Daphnia* was chosen as the test target since it is one of the indicator species of zooplankton used for monitoring aquatic ecosystems and has a strong swimming ability. *Daphnia* could swim freely in the water, which was similar to a natural water environment. A preliminary research study on *Daphnia* revealed three distinct swimming behaviors: fast swimming, moderate swimming, and sinking [38,39]. Typical swimming speeds for *Daphnia* range between 3 and 8 mm s^−1^. The exposure time of the light source was set as 1 ms to eliminate the motion blur and provide sufficient light to capture the fast-moving objects. The image quality and image definition of underwater images were evaluated based on the captured *Daphnia* images. Several images of *Daphnia* captured by the underwater imaging system are presented in Figure 12.

The captured images show that the *Daphnia* individuals are semi-transparent with visible internal structures and present a high variety of body shapes and sizes. These *Daphnia* images have a high contrast and high definition. The detailed features of *Daphnia*, e.g., body contours, textures, and antennae, are accurately recorded, regardless of the swimming speed and locations in the field of view of the camera. These results prove that the light source produced uniform illumination, ensuring that clarity and contrast were maintained in the whole image. Motion blur did not occur in the *Daphnia* images. The captured images had a high definition and presented detailed features of the plankton. Therefore, they could provide sufficient image features for the automated species identification system developed for plankton.

**Figure 13:**
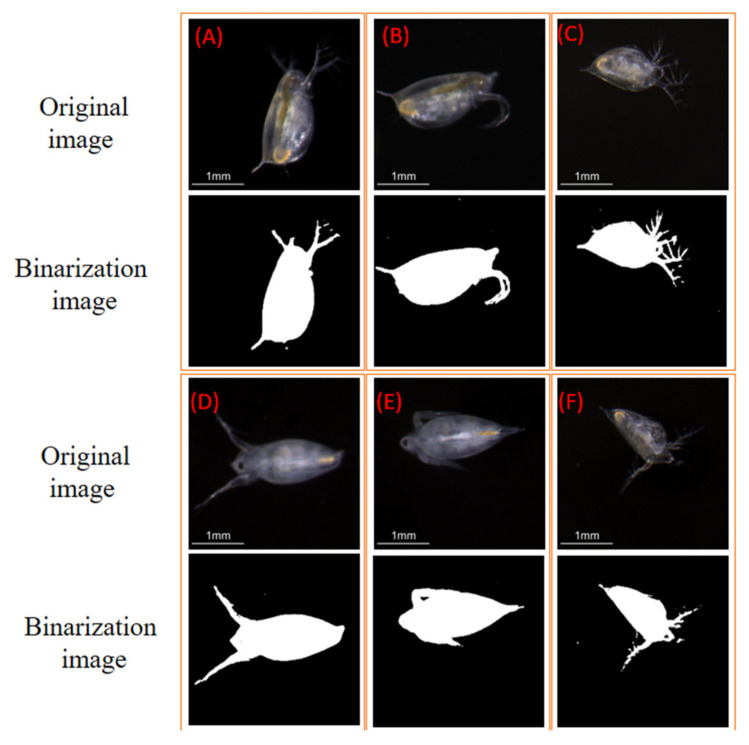
The original images and binarized images of zooplankton in the ROIs (**A**–**F**).

Since uniform background illumination and a high contrast over the field of view were achieved, the image noise was maximally depressed in the results and almost dark backgrounds were observed in the captured images. Consequently, the *Daphnia* images could be accurately extracted from the background without sophisticated algorithms. A classical global thresholding algorithm, OTSU, was employed to segment the *Daphnia* individuals. A binary image based on setting a threshold value on the pixel intensity of the original image was created to separate the foreground pixels from background pixels. The segmented results are presented in the binary images below the original images in Figure 13. The boundaries of the binary images are identical to the actual shape of *Daphnia*. Additionally, no artifacts appeared in these images, which are usually caused by non-uniform illumination.

After the segmentation, the geometric features of *Daphnia* could be calculated from the binary image. Five common features, including the area size, major axis length, minor axis length, equivalent diameter, and perimeter, were measured. Moreover, the gray-level intensity of the *Daphnia* individuals was calculated as the brightness feature. The measurement results of *Daphnia* from Figure 13A–F are listed in Table 1. The precision level of the measured features is given in micrometers. Tiny antennae with diameters of <80 μm were able to be identified in all figures. In the binary images, the silhouettes of *Daphnia* are roughly ellipsoidal. Consequently, we chose to represent each individual by the major and minor axes of an ellipse fitted to the outline of its silhouette. Image analysis also provides a rapid and accurate determination of the area, volume, or biomass distribution and life-table information of *Daphnia*.

When the images of *Daphnia* swimming in a chamber were randomly obtained, the area of *Daphnia* varied, depending on the position and direction of the *Daphnia* [39]. As *Daphnia* typically swim at oblique angles, the length of silhouette projections on the horizontal plane will underestimate the true body length. However, the minor axis (body width) will be correctly imaged, irrespective of the swimming angle. As the length of *Daphnia* changes discontinuously at molting, while the width changes continuously with body mass, the body width should be more sensitive to the nutritional status than the body length [40]. Therefore, we could use the width axis rather than the total length to evaluate the growth of *Daphnia.* Moreover, the pictures also showed the transparency of *Daphnia*’s body and internal organs. The gut of a *Daphnia* consists of three parts—the esophagus, midgut, and hindgut—and is more or less tubular. The color of *Daphnia* adapts to the food that is predominant in their diet [41]. It can be clearly seen that *Daphnia* is transparent with a tint of green or yellow because of feeding on green algae.

**Figure 14:**
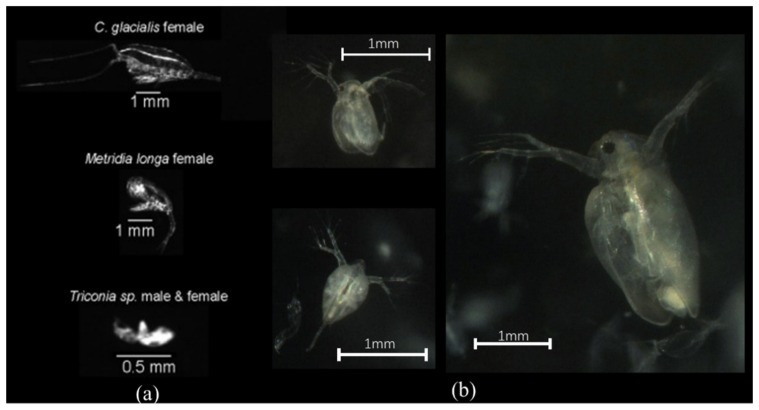
Comparison with LOKI. (**a**) Images captured by LOKI [11]; (**b**) images captured by the proposed system.

Furthermore, the proposed underwater imaging system was compared with the LOKI system. Figure 14 presents images of biological objects captured by LOKI and the proposed system. The LOKI system has a large field of view and could capture the large size of objects. However, color information was lost in the LOKI system. Additionally, small-sized objects could not be accurately recorded, especially when the object was <1 mm (Figure 14a). Our proposed system could capture very detailed body parts (e.g., antennae) in colored images. For the semi-transparent *Daphnia*, the organs in the body were able to be clearly presented (Figure 14b). In this work, the tests were carried out in simulated conditions in the laboratory which has few noises comparing with in situ observation, such as the results from LOKI system [11]. In the further, our developed imaging system will be deployed in coastal area.

**Figure 15:**
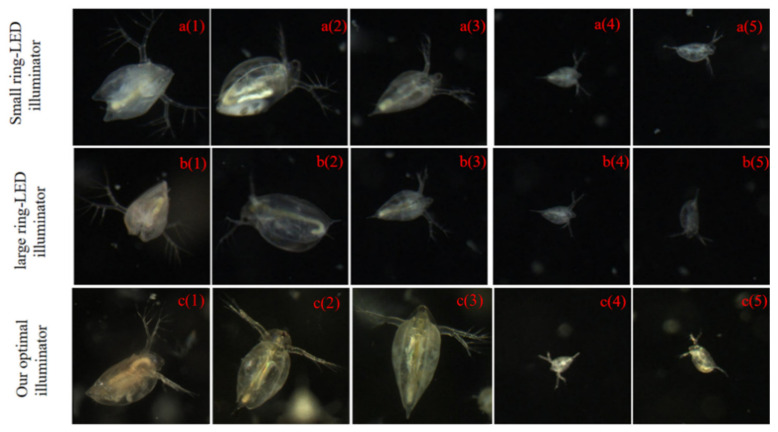
Images of *Daphnia* captured by the imaging system with the small ring-LED illuminator (**a1**–**a5**), the large ring-LED illuminator (**b1**–**b5**), and the optimal illuminator (**c1**–**c5**).

In order to evaluate the effect of the optimal illumination on the imaging performance, we considered both common illumination cases—a small ring-LED illuminator (the model code: JS-RL-100-00-W, YUAND Tech, Shenzhen, China) and a large ring-LED illuminator (the model code: JX-RX-120-00-W, YUAND Tech, Shenzhen, Country)—in our designed light source system. The small ring-LED illuminator consists of 49 LED lamps, with an outer and inner diameter of 100 and 70 mm, respectively. The large ring-LED illuminator consists of 63 LED lamps, with an outer and inner diameter of 120 and 90 mm, respectively. The imaging system was placed in a fixed position near the center lighting position. The zooplankton sample was living *Daphnia*. Images were collected under three different lighting conditions. We selected five groups of pictures with a similar growth status and swimming posture from the three imaging data sets. The montage in Figure 15 clearly illustrates the various illumination modalities and the corresponding images generated by the imaging system.

As can be seen from these figures, the texture features of the *Daphnia* appear different under different lighting conditions. The brightness of images was higher under optimized lighting conditions compared to the commercial illuminators.

In order to obtain a quantitative comparison, we analyzed the texture features (Table 2) described in the zooplankton images: energy, contrast, entropy, and homogeneity. Energy reflects the grayscale distribution homogeneity of images and texture crudeness [42]. Contrast measures the degree of texture smoothness, which is low when the image has constant gray levels. Entropy reflects the degree of disorder in an image. Homogeneity reflects the homogeneity of image textures and scales the local changes of image texture, which is high in the absence of intra-regional changes and has a locally homogenous distribution in image textures [43].

The results of the calculation can be seen in Table 3. The evaluation of texture features of the zooplankton image is consistent with human visual perception. With our illumination system, the values of contrast and entropy are higher, and the values of energy and homogeneity are lower. The proposed optimal illumination scheme provides richer texture feature information of zooplankton images than the commercial illuminators. The experiment verified the effectiveness of the optimal illumination scheme, and the image performance was also improved by a better contrast and clear image.

**Figure 16:**
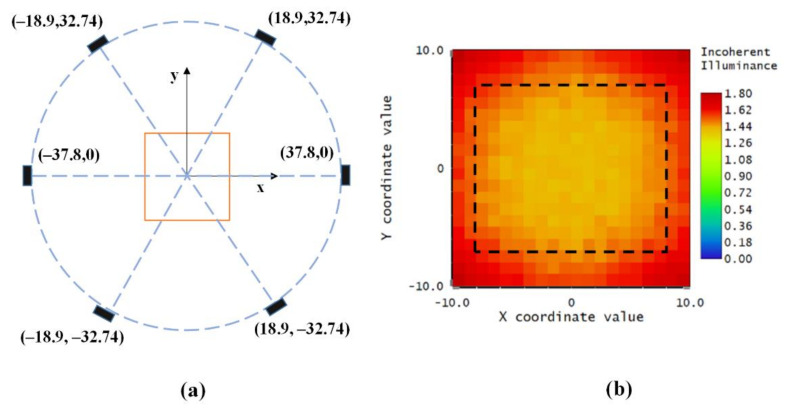
(**a**) The geometric arrangement of the simple LEDs array; (**b**) Irradiance distribution over the target plane of the simple LEDs array. Dashed rectangle indicates the imaging area of the camera.

In addition, a comparison test on optimized lighting configuration and equally placed lighting configuration was conducted. The six LEDs in the equal lighting configuration were equally spaced apart in a circle arrangement. The radius was the same with the optimized configuration (i.e., 37.8 mm). The geometric arrangement of the equally placed LED array and its irradiance map are presented in Figure 16. The dashed rectangle represents the field of view of the camera in the Figure 16b. As can be seen from the irradiance map, the illumination uniformity is low in the four corners and border area in the target plane. By analyzing the illuminance distribution pattern of our proposed configuration (Figure 9b) and the equally spaced configuration (Figure 16b), the optimized lighting configuration shows better uniformity than the equally spaced one.

**Figure 17:**
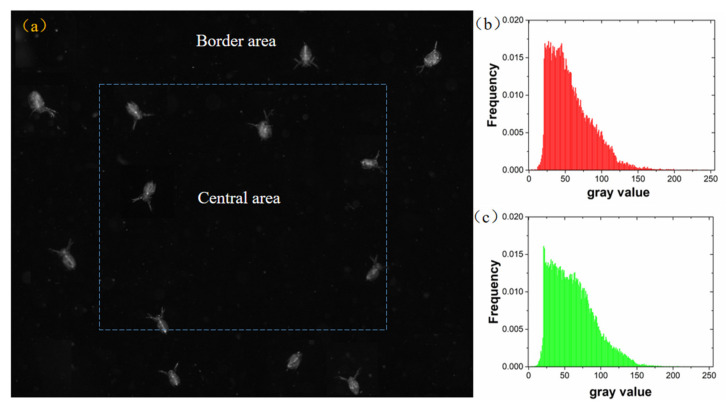
(**a**) The division of *Daphnia* image under equally spaced lighting configuration; (**b**) The normalized histogram of *Daphnia* images in the central area; (**c**) The normalized histogram of *Daphnia* images in the border area.

Subsequently, the two lighting systems were tested on imaging living *Daphnia*. To examine the uniformity and image quality the whole image was divided into central area and border area (Figure 17a). The *Daphnia* images located in the central area and border area were extracted and their grey histogram was calculated, separately. A normalized histogram of each *Daphnia* images was constructed. Average normalized histograms for *Daphnia* images in the central and border area are presented in Figure 17b,c. The distribution of histograms of the central images showed different patterns with the histograms of border images. The mean gray values of *Daphnia* images were calculated. The average of the mean gray values of the *Daphnia* images in the central region is 57.6, and the mean gray values of the *Daphnia* images in the edge region is 61.8. These results show that *Daphnia* images in central area more pixels in low value which might indicate light intensity is lower than border area.

**Figure 18:**
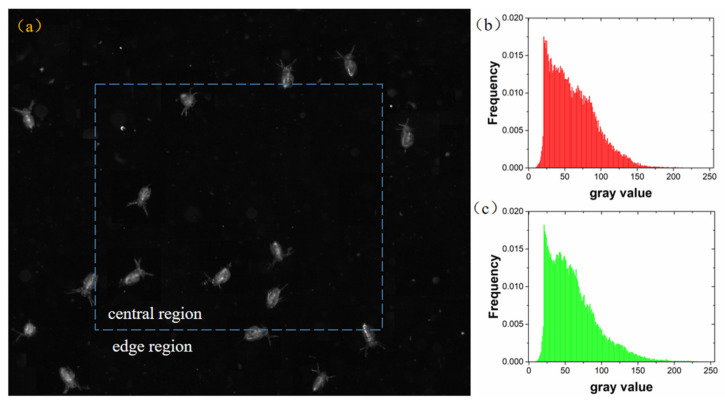
(**a**) The division of *Daphnia* image under optimal lighting configuration; (**b**) The normalized histogram of *Daphnia* images in the central area; (**c**) The normalized histogram of *Daphnia* images in the border area.

Accordingly, Daphnia images captured by our optimized configuration was analyzed in the same manner (Figure 18). The average normalized histogram of *Daphnia* images in central and border area are presented in Figure 18b,c. The image in central area and border area presented similar pattern in histogram. The experiments showed that the mean gray values of the *Daphnia* images in the central and border area was 61.7 and 61.9, respectively. Therefore, the gray value distribution shows that the illumination difference between central and border area was relatively small under the optimized illumination condition. The imaging brightness of the organism is close to the same level at different positions in the camera’s field of view, which can provide more accurate images for further analysis.

The underwater imaging system was established based on the optimized illumination configuration of an LED array. The power consumption of our imaging system was measured in laboratory conditions. The maximal power consumption was 7.89 Watts and the average power consumption was 7.4 Watts during capturing. Moreover, the stand-by power consumption was approximately 6.24 Watts. The power consumption was affordable by batteries.

Therefore, the underwater plankton imaging system developed not only provides high-resolution images and precise analysis for zooplankton, but also gives accurate information on the growth state of living individuals. This system is promising for high-throughput *Daphnia* toxicity tests and real-time morpho-physiological trait observations. The imaging scale could also be refined to increase the capability to observe smaller types of zooplankton. Automated species identification should be integrated into the developed system for in situ real-time plankton monitoring. In addition, our system can also be deployed from ships or in tow. To obtain a fine image of zooplankton in open water imaging, we need to further consider the velocity of the water relative to the camera. The minimum exposure time is 0.1 ms, which is enough to eliminate the motion blur at the speed of 0.15 m/s and ensure that sufficient light reaches the camera.

## 4. Conclusions

This work presents a novel method for optimizing the LED array of a dark-field illumination system for in situ zooplankton imaging. The proposed imaging system was simple to build, is cost-effective, and has low power consumption. The effects of multiple LEDs on the illumination uniformity were investigated by analyzing various configurations and the packaging density of an LED array. The genetic algorithm was applied to optimize the LED array for a uniform illumination distribution. The results proved that the illumination system and imaging system are designed to have a compact size and low power consumption, which means that they are very suitable for being deployed on underwater observation platforms for zooplankton monitoring. By comparing the proposed system with another underwater zooplankton imaging system, the developed imaging system could further optimize the design for the underwater illumination system and could provide high-resolution and high-definition images for the in situ accurate measurement of zooplankton. This work demonstrated the theoretical and preliminary experimental results of a cost-effective underwater imaging system with dark-field illumination. The developed zooplankton imaging system could be the key component for the automatic monitoring of marine biological organisms in real-time. This work is beneficial for the practical applications of mesoplankton detection, and also provides a reference for future studies of other underwater lighting and imaging systems.

In future works, our zooplankton imaging system can be deployed with multiple environment sensors (such as a CTD, an oxygen optode, and a speed meter) on a single platform. The developed platform could be used to study the fine-scale behaviors of marine organisms and record the key parameters of their surrounding physical and chemical environment in real-time. This could help investigators advance their understanding of ocean biogeochemistry, biology, and ecosystems.

## Figures and Tables

**Table 1:** The measurement results of zooplankton.

Parameters	A	B	C	D	E	F
Area (mm)	1.663	1.673	1.177	1.626	1.526	1.080
Major axis length (mm)	2.382	2.308	1.856	2.504	2.055	1.671
Minor axis length (mm)	1.040	1.076	0.928	1.122	1.012	1.205
Equivalent diameter (mm)	1.455	1.459	1.198	1.439	1.394	1.173
Perimeter (mm)	9.778	7.789	9.633	9.770	6.473	8.692
Mean of brightness	95.04	76.24	73.94	83.99	78.93	68.44
Standard deviation of brightness	42.68	34.77	40.75	37.02	35.03	35.10

**Table 2:** The texture features used in this study.

Method	Formula
Energy	$\sum_{i,j=1}^{N_{g}}P_{ij}^{2}$
Contrast	${\sum_{i,j=1}^{N_{g}}P_{ij}(i-j)}^{2}$
Entropy	$-\sum_{i,j=1}^{N_{g}}P_{ij}\log P_{ij}$
Homogeneity	$\sum_{i,j=1}^{N_{g}}\frac{P_{ij}}{1+|i+j|}$

**Table 3:** Texture characteristic parameter computed result.

Images	Energy	Contrast	Homogeneity	Entropy
**a1**	0.6478	0.0270	0.9866	4.6817
**b1**	0.6663	0.0291	0.9857	5.1094
**c1**	0.5906	0.0359	0.9825	5.3417
**a2**	0.6184	0.0368	0.9823	4.7674
**b2**	0.6600	0.0168	0.9916	4.4639
**c2**	0.6842	0.0738	0.9738	4.9676
**a3**	0.7594	0.0162	0.9919	4.0554
**b3**	0.8475	0.0103	0.9948	3.6599
**c3**	0.6181	0.0651	0.9727	4.8811
**a4**	0.9283	0.0054	0.9973	2.8527
**b4**	0.9375	0.0054	0.9973	3.1140
**c4**	0.9381	0.0145	0.9946	3.4211
**a5**	0.9191	0.0099	0.9951	3.4720
**b5**	0.9376	0.0046	0.9977	2.9578
**c5**	0.8881	0.0190	0.9923	3.9563
